# Wide Diversity of Coronaviruses in Frugivorous and Insectivorous Bat Species: A Pilot Study in Guinea, West Africa

**DOI:** 10.3390/v12080855

**Published:** 2020-08-05

**Authors:** Audrey Lacroix, Nicole Vidal, Alpha K. Keita, Guillaume Thaurignac, Amandine Esteban, Hélène De Nys, Ramadan Diallo, Abdoulaye Toure, Souana Goumou, Abdoul Karim Soumah, Moriba Povogui, Joel Koivogui, Jean-Louis Monemou, Raisa Raulino, Antoine Nkuba, Vincent Foulongne, Eric Delaporte, Ahidjo Ayouba, Martine Peeters

**Affiliations:** 1 TransVIHMI, Institut de Recherche pour le Développement, University of Montpellier, INSERM, 34394 Montpellier, France; audrey.lacroix@ird.fr (A.L.); nicole.vidal@ird.fr (N.V.); alpha-kabinet.keita@ird.fr (A.K.K.); guillaume.thaurignac@ird.fr (G.T.); amandine.esteban@ird.fr (A.E.); abdoulaye.toure@insp-guinee.org (A.T.); raisa.raulino@ird.fr (R.R.); antoine.nkuba@ird.fr (A.N.); eric.delaporte@ird.fr (E.D.); 2 Centre de Recherche et de Formation en Infectiologie de Guinée (CERFIG), Université Gamal Abder Nasser de Conakry, Conakry BP6629, Guinea; souana.goumou@cerfig.org (S.G.); abdoul.soumah@cerfig.org (A.K.S.); moriba.povogui@cerfig.org (M.P.); joel.koivogui@cerfig.org (J.K.); jean.monemou@cerfig.org (J.-L.M.); 3 ASTRE, CIRAD, INRA, University of Montpellier, 34398 Montpellier, France; helene.de_nys@cirad.fr; 4 CIRAD, UMR ASTRE, Harare, Zimbabwe; 5 Laboratoire Central de Diagnostic Vétérinaire, Ministère de l’Elevage et des Productions Animales, Conakry BP3982, Guinea; dialloramadan1954@gmail.com; 6 Institut National de Sante Publique (INSP), Conakry BP6623, Guinea; 7 Institut National de Recherche Biomédicale and Service de Microbiologie, Cliniques Universitaires de Kinshasa, Gombe, Kinshasa P.O. Box 1197, Democratic Republic of the Congo; 8 Département de Bacteriologie-Virologie, CHU de Montpellier, 34295 Montpellier, France; v-foulongne@chu-montpellier.fr

**Keywords:** bat, coronavirus, Guinea, virus diversity, Africa

## Abstract

Zoonoses can constitute a threat for public health that can have a global importance, as seen with the current COVID-19 pandemic of severe acute respiratory syndrome coronavirus (SARS-CoV2). Bats have been recognized as an important reservoir of zoonotic coronaviruses (CoVs). In West Africa, where there is a high diversity of bat species, little is known on the circulation of CoVs in these hosts, especially at the interface with human populations. In this study, in Guinea, we tested a total of 319 bats belonging to 14 genera and six families of insectivorous and frugivorous bats across the country, for the presence of coronaviruses. We found CoVs in 35 (11%) of the tested bats—in three insectivorous bat species and five fruit bat species that were mostly captured close to human habitat. Positivity rates varied from 5.7% to 100%, depending on bat species. A wide diversity of alpha and beta coronaviruses was found across the country, including three sequences belonging to SarbeCoVs and MerbeCoVs subgenera known to harbor highly pathogenic human coronaviruses. Our findings suggest that CoVs are widely spread in West Africa and their circulation should be assessed to evaluate the risk of exposure of potential zoonotic CoVs to humans.

## 1. Introduction

Emerging infectious diseases (EIDs) represent a significant challenge to global health. The frequency of EID outbreaks has dramatically increased over the last decades. Most EIDs have a zoonotic origin; more than 70% of them are caused by pathogens originating from wildlife [1]. The continuing encroachment of human populations into areas initially only occupied by wildlife, such as pristine forest—together with environmental and climatic changes—has led to increasing contacts between humans and animals and as a consequence, exposure to the pathogens they harbor [2,3,4]. The ongoing outbreak with the new severe acute respiratory syndrome coronavirus (SARS-CoV-2), responsible for the COVID-19 pandemic, is a perfect illustration of the potential impact and the global consequences resulting from a single cross-species transmission from an animal virus. The epidemic was first recognized in December 2019 in China and has spread across the entire globe in a few months. By the end of May 2020, more than five million people have been infected; more than 350,000 have died and a lockdown of four billion people across the globe has been enforced to reduce the spread of the virus [5]. It is thus important to be prepared for new EID outbreaks. A major step in understanding the risk for zoonotic infections, is to characterize the diversity of pathogens at the interface between humans and animals. Knowing the animal sources, prevalence, geographic distribution and the genetic diversity or evolutionary history of pathogens in wildlife is critical to evaluate the risk for potential emergence or reemergence of disease.

The current COVID-19 outbreak with the SARS-CoV-2 virus, is the seventh coronavirus (CoV) identified in humans since the 1960s [6,7]. Whereas the initial known human CoVs (HCoV-229E, HCoV-OC43, HCoV-HKU1 and HCoV-NL63) usually cause mild symptoms, the more recent HCoVs have higher pathogenicity and impact. SARS-CoV-1 emerged in 2003, infecting 8000 people in more than 30 countries in a few months, with a lethality of 10% [8,9]. MERS-CoV emerged in 2012, causing more than 2500 cases in 27 countries to date, with a lethality of 35% [10,11,12]. The recent SARS-CoV-2 outbreak has an estimated lethality that is lower than SARS-CoV-1, but the virus was able to spread extremely fast worldwide [13]. All seven HCoVs have a zoonotic origin and evolutionary origin from bats [14,15,16,17]. Some have been directly transmitted from bats to humans, but for others an intermediate host was required, like camels for MERS-CoV or civets for SARS-CoV-1 [18,19,20,21,22,23]. For SARS-CoV-2, the closest relative is identified in a bat from China and the role of an intermediate species, i.e., pangolins, is not clarified yet [24,25].

It is now well established that CoVs are widespread in bats with relative high prevalence and important genetic diversity providing thus multiple opportunities for the potential emergence of novel HCoVs in areas where contact between humans and bats are frequent [26]. Nevertheless, despite recent efforts, our knowledge on CoVs in bats is far from complete, because most studies are conducted on a limited number of species and in restricted geographic or ecological environments. Bats represent 20% of all mammals with more than 1400 different species [27]. More studies are thus still needed, especially in areas where the risk for spillover to animals or humans is high, like southeast Asia or West and Central Africa, where numerous bat species are hunted for food or medicine [28,29,30]. Moreover, bushmeat hunting is recognized as a major threat for conservation of bats in these areas [31,32]. In addition, humans can also be indirectly exposed to bat viruses through contacts with bat guano or fruit contaminated by their saliva, urine or feces [32,33].

Several studies have shown a large diversity of CoVs in bats across Africa, but limited data are currently available on bat CoVs in Sub-Saharan Africa and especially from West Africa, where we have currently only information on bats from Ghana and Nigeria [15,34,35,36,37,38,39,40,41,42]. Nevertheless, new alpha (α-CoVs) and betacoronaviruses (β-CoVs) have been described in Africa, including some closely related to human CoVs such as HCoV-229E, or SARS-CoVs [15,34,35]. Recently, the closest relatives in bats to the highly pathogenic MERS-CoV were documented in *Neoremicia* and *Pipistrellus* bats from South-Africa and Uganda, respectively, illustrating that African bats could also play a role in outbreaks of CoVs in humans [23,43]. Here we conducted a pilot study on the presence of CoVs in bats in Guinea, West Africa, a country with a high likelihood for EID outbreaks and where humans are frequently directly or indirectly exposed to bats (A.K. Keita, personal observation).

## 2. Materials and Methods

### 2.1. Study Sites, Sample Collection and Ethical Permits

Samples were collected from free-ranging frugivorous and insectivorous bats in Guinea between February 2016 and January 2017 as previously described [44]. We obtained permission to conduct research and to collect samples from the National Ethics Committee for Health Research (CNERS) from Guinea (approval reference 074/CNERS/15, 26 November 2015). Briefly, bats were captured using mist nets or harp traps in roosting and foraging sites. Bats were then kept in cotton bags and released immediately after sampling. Whole blood was taken by venipuncture of the propatagial or brachial vein and blood drops were directly transferred onto Whatman 903 filter paper (GE-Healthcare, Feasterville-Trevose, PA, USA). Samples were air-dried and preserved individually in plastic bags containing silica desiccant and stored in a hermetic box as dried blood spots (DBS). DBS were stored at −40 °C in the laboratory (2 to 3 weeks after collection) and kept frozen until analysis. Rectal and oral swabs were also collected in 500 µL RNA-later to preserve RNA from degradation and stored in the field at ambient temperature for maximum two weeks and subsequently at −40 °C in the laboratory. For a subset of animals, fresh fecal samples were also collected and stored in RNA-later, i.e., feces dropped when bats were in cotton bags. For each sampled bat, information on capture sites (GPS coordinates, ecological environment), capture method, morphology (body measurements, weight, color), sex, age class (adult, juvenile) and visual species identification were recorded.

### 2.2. Nucleic Acid Extraction

Total DNA and RNA were extracted from swabs and feces using the NucliSENS EasyMAG platform (BioMérieux, Marcy-l’Etoile, France). Briefly, 250 µL of sample was incubated with 2 mL of lysis buffer for 15 min and extraction was performed using manufacturer’s instructions. Total nucleic acids were resuspended in 60 µL elution buffer.

### 2.3. Molecular Confirmation of Bat Species

Species identification recorded in the field was molecularly confirmed on a subset of bats using DBS as in our previous study on Ebolaviruses in bats [44]. In addition, the species was also molecularly confirmed for each bat in which a coronavirus was identified. A fragment of approximately 800 base pairs (bp) of the mitochondrial cytochrome b (CytB) region was amplified using previously described primers to identify mammal species including bats, Cytb-L14724 (forward) and Cytb-H15506 (reverse), [45]. However, the forward primer was replaced by a newly designed primer Cytb-L1 5′- ATG ACC AAC ATC CGA AAA TCN CAC-3′ or Cytb-L2 5′- ATY TCY TCM TGA TGA AAY TTY GGM T- 3′ to increase PCR specificity for certain bat species. PCR products were diluted and directly sequenced with BigDye Terminator version 3.1 sequencing kit (Life Technologies, Courtaboeuf, France). Electrophoresis and data collection were processed on an Applied Biosystems 3500 Genetic Analyzer (Thermo Fisher Scientific, Foster City, CA, USA). Sequences from both strands were reconstituted using SeqMan Pro tool from the package DNAStar v17.0.2 (Lasergene, Madison, WI, USA). Sequences were pasted in the NCBI BLAST web interface (https://blast.ncbi.nlm.nih.gov/Blast.cgi) to identify the most similar bat species. For samples with no or low similarity (<97%) hits with species in Genbank, a phylogenetic tree was constructed using maximum likelihood methods implemented in PhyML with reference sequences in order to obtain genus identification. The new sequences were deposited in the GenBank under accession numbers MT586773-MT586805.

### 2.4. RT–PCR Screening for Detection of Coronavirus RNA

First, cDNA was synthesized from denatured RNA (70 °C for 10 min) using a Reverse Transcription System kit with random primers (Promega, Madison, WI, USA), following manufacturer’s instructions. PCR screening was done with a broadly reactive nested PCR approach in the highly conserved RdRp region. Our assay targeted a 440 bp fragment using degenerate primers from a modified protocol described by Chu et al. (2011). In the first round, a 602 bp fragment was targeted following previously described PCR methods for which amplification conditions were slightly adapted [46]. Briefly, cDNA was amplified using the GoTaq Hot Start Master Mix PCR kit (Promega, Madison, WI, USA) as follows: 40 cycles of 92 °C for 30 s, 48 °C for 30 s and 72 °C for 50 s for the first round of PCR. In the second round, a 440 bp product was amplified and cycling conditions were modified for the second round of PCR, using a touch-down technique to reduce nonspecific amplifications (10 cycles of 92 °C for 30 s, 53 °C for 30 s with −0.5 °C/cycle and 72 °C for 30 s, 35 cycles of 92 °C for 30 s, 53 °C for 30 s and 72 °C for 30 s). PCR products were first analyzed by agarose gel electrophoresis and positive amplicons were sequenced as indicated above and analyzed on the Applied Biosystems 3500 genetic analyzer platform. Sequences from both strands were reconstituted using SeqMan Pro tool from the package DNAStar v17.0.2 (Lasergene, Madison, WI, USA). Sequences were pasted in the NCBI BLAST web interface (https://blast.ncbi.nlm.nih.gov/Blast.cgi) to identify similar coronavirus sequences. The PCR assay was first tested on six nasopharyngeal swabs from patients positive for SARS-CoV-2, which are anonymous and untraceable diagnostic leftover samples, kindly provided by the Virology department of Montpellier University Hospital in March 2020. Amplification products and sequences were obtained, demonstrating that the assay is also able to detect the novel SARS-CoV-2 strain (data not shown).

### 2.5. Phylogenetic Analyses

The new sequences were aligned with representatives of alpha and beta-coronavirus sequences that have been reported mainly in Africa and elsewhere (Table S1). A multiple sequence alignment (MSA) was obtained by using MAFFT v7 (https://mafft.cbrc.jp/alignment/server/). The alignment was manually checked and end-trimmed to match with the newly obtained RdRp sequences and to remove the PCR primers sequences. The final MSA was used for maximum likelihood (ML) phylogenetic analysis with GTR + F + I + 4Γ as the best-fit model of nucleotide substitution according to BIC and 1000 bootstrap resampling by using IQ-Tree server (http://iqtree.cibiv.univie.aC.at) [47,48]. Consensus trees were edited with FigTree v1.4.4. The new sequences were deposited in GenBank under accession numbers MT586830-MT586867.

### 2.6. Statistical Analyse

To explore the impact of age, sex and environment of the collected samples on the detection of coronavirus RNA, we performed Kruskal–Wallis test on bat field data. Significant results were considered for a *p*-value of <0.05.

## 3. Results

### 3.1. Bat Species and Sampling

**Figure 1:**
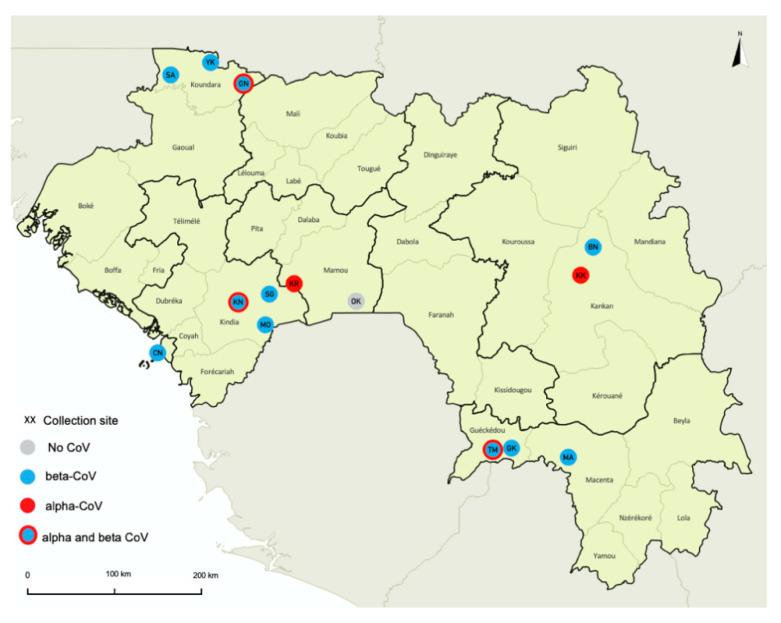
Bat collection sites in Guinea. Sites where samples from bat were collected are highlighted with circles on the map as follows: gray indicates site where no coronavirus (CoV) was detected; red, sites where alphacoronavirus (α-CoVs) were detected; blue, sites where betacoronavirus (β-CoV) were detected, blue and red, sites where both α-CoVs and β-CoVs were detected. Sites are abbreviated as follows: for the prefecture of Conakry: CN—Conakry; for the prefecture of Kindia: KN—Kindia; MO—Madina Oula; SG—Souguéta; for the prefecture of Kankan: KK—Kankan; BN—Baté-Nafadji; for the prefecture of Gueckedou: TM—Termessadou-Dibo; GK—Tékoulo; for the prefecture of Koundara: GN—Guingan; SA—Sareboido; YK—Youkounkoun; for the prefecture of Mamou: KR—Konkouré; OK—Ouré-Kaba; and for the prefecture of Macenta: MA—Macenta.

Samples from a total of 319 wild bats, captured at 14 sites in seven different prefectures in Guinea, were studied (Figure 1, Table 1). Bats were captured in different ecological settings including the following environments: villages (38.6%), forest sites (30.1%), urban sites (23.2%), plantations (4.1%) and diverse other settings for the remainder (4.1%). Species identification was confirmed by CytB sequence analysis on a total of 171 (53.6%) bats to confirm at least one sample per sampling date, per capture method and per morphologic description at each site. Species identification was then extrapolated for the remaining samples by combining molecular and field data. For certain insectivorous bats (Molossidae, Rhinolophidae, Hipposideridae and Nycteridae) identification was only possible at the genus level, mostly due to the lack of reference sequences in Genbank. Because discrimination between *Epomophorus gambiensis* and *Micropteropus pusillus* was not possible based on CytB sequences only, we used morphologic details on forearm and weight measurements to discriminate the species for 93 bats, as previously documented by others [49]. Details on bat families, genera and species are shown in Table 2. We collected samples from 274 (85.9%) frugivorous bats (Pteropodidae family) representing at least eight species and 45 (14.1%) insectivorous bats from five families and at least six genera. Overall, 195 (61.1%) bats were female, 121 (37.9%) were male and for three (1.0%) sex classification was not available. The vast majority were adults (300; 94.1%), 11 (3.4%) were juveniles and for eight bats (2.5%), age could not be identified, or information was not recorded.

### 3.2. PCR Screening and Presence of α and β Coronaviruses

We screened a total of 634 samples from 319 bats by RT–PCR for the presence of coronavirus RNA: for 286 (89.7%) bats oral and rectal swabs were tested, 19 (6.6%) bats were tested using oral swabs and fecal samples, for five (1.6%) oral and rectal swabs and feces, for two and seven bats only rectal or oral swabs were tested, respectively. A total of 38 samples from 35 of the 319 tested bats (11%), were positive for the presence of CoVs; five in oral swabs only, 20 in rectal swabs only, seven in feces only, one in both feces and oral swab, two in rectal and oral swabs (Table 3). The rate of positivity varied according to the sample type; the proportion of positive samples was 7.5% (22/293) in rectal swabs, 2.5% (8/317) in oral swabs and 33.3% (8/24) in fresh fecal samples. Among the three bats that were positive in two different samples, two were infected with the same virus in the rectal and oral swab or in feces and oral swab, but for one bat, an α-CoV and a β-CoV were detected in oral and rectal swab, respectively.

We confirmed the presence of CoVs in 10/45 (22.2%) insectivorous bats from three species belonging to three families, i.e., Hipposideridae (33.3%, 7/21), Rhinolophidae (40.0%, 2/5) and Nycteridae (33.3%, 1/3). Among the frugivorous bats, 25/274 (9.1%) tested positive and CoVs were detected in five species: 4/9 (44.4%) *Eidolon helvum*, 12/120 (10.0%) *Rousettus aegyptiacus*, 3/42 (7.1%) *Lissonycteris angolensis*, 5/87 (5.7%) *Epomophorus gambianus* and the single *Nanonycteris* sp. bat. CoVs were identified in all provinces and in almost all sites where bats were sampled in Guinea (Figure 1). Among the positive bats, 72% were sampled close to human habitats, 23% in urban areas and 49% in villages. No significant impact of the age, sex of bats or environment on the presence of CoV was detected, using Kruskal–Wallis test (*p*-values > 0.05).

We identified thus 38 CoV sequences, 15 sequences belonging to the α-CoV genus and 23 to the β-CoV genus, the latter known to harbor five HCoVs, i.e., HCoV-OC43, HCoV-HKU1 and the highly pathogenic SARS-CoV-1, MERS-CoV and the recently described SARS-CoV-2 from the ongoing COVID-19 pandemic. Of all, 14 bats were infected with α-CoVs, 20 with β-CoV and one bat (*R. aegyptiacus*) was co-infected with both, as mentioned above. Among the 15 α-CoVs, six were detected in oral swabs only, one in oral and rectal swabs, one in feces and oral swabs, four in rectal swabs and three in feces only. All 23 β-CoVs were identified in rectal swabs or feces and not in oral swabs.

### 3.3. Genetic Diversity of Bat Coronaviruses

**Figure 2:**
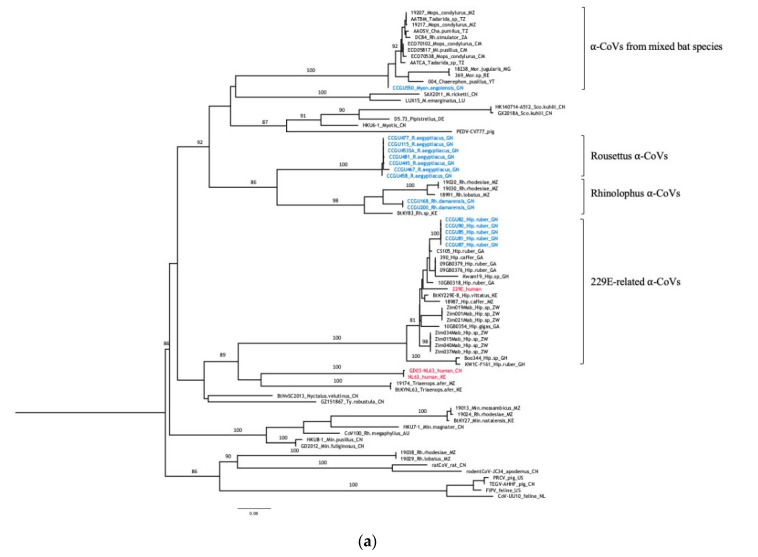

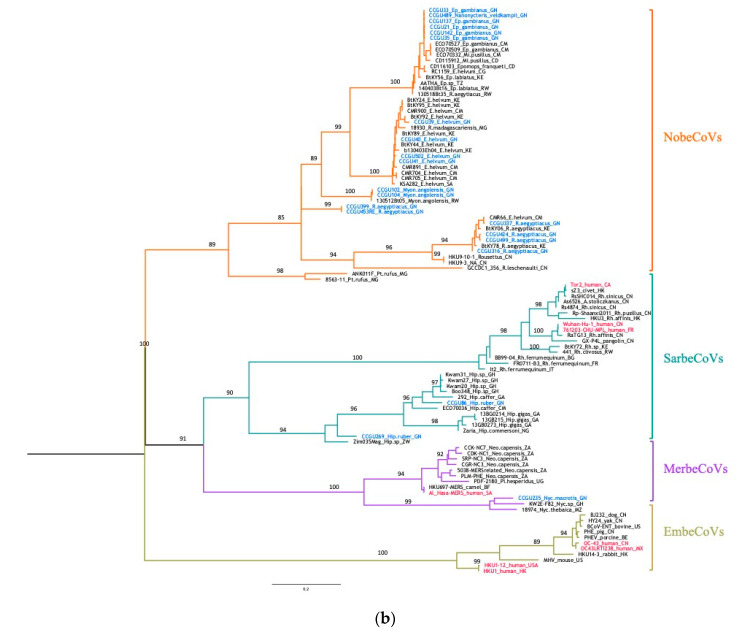
Maximum likelihood (ML) consensus trees derived from coronavirus sequences in the RNA-dependent RNA-polymerase partial nucleotide sequences (374 unambiguously aligned base pairs). (**a**) consensus tree derived from 78 alphacoronaviruses; (**b**) consensus tree derived from 102 betacoronaviruses. Phylogenetic tree analysis was performed using the GTR + F + I + 4Γ nucleotide substitution model and 1000 bootstrap resampling. Sequences in blue refer to the new bat CoVs detected in this study. Human CoVs are highlighted in red. Branch supports >0.75 are indicated on the trees. Trees were generated as indicated in Material and Methods and edited with increasing nodes and midpoint rooting in FigTree. Details on reference sequences are provided in Supplementary Table S1. Abbreviations of bat genera are as follows: Ep—*Epomophorus*; R—*Rousettus*; E—*Eidolon*; Myon—*Lissonycteris (previously Myonycteris)*; Mi—*Micropteropus*; Nyc—*Nycteris*; Hip—*Hipposideros*; Rh—*Rhinolophus*; Neo—*Neoromicia*; Pt—*Pteropus*; A—*Aselliscus*; Pi—*Pipistrellus*; Min—*Miniopterus*; Mor—*Mormopterus*; ChA—*Chaerephon*; M—*Myotis*; Sco—*Scotophilus*.

The phylogenetic analysis based on 374 bp nucleotides of the new sequences and reference sequences is shown in Figure 2. High genetic diversity was observed among the newly identified α and β-CoVs. α-CoVs were detected in 7/45 (15.6%) of insectivorous bats; five *Hipposideros* sp. and two *Rhinolophus* spp. (Figure 2A). All α-CoV strains from *Hipposideros* bats in Guinea clustered with previously reported CoV sequences from bats from this genus in Zimbabwe, Gabon or Ghana and comprising also the HCoV-229E virus. The new α-CoV strains from *Rhinolophus* bats fall in a cluster that groups sequences from other *Rhinolophus* bats captured in Mozambique and Kenya. Among frugivorous bats, 8/274 (2.9%) were infected with α-CoV; seven *R. aegyptiacus* and one *L. angolensis*. α-CoVs from *R. aegyptiacus* formed a separate lineage for which no close relatives (>90% homology) were detected in Genbank. The new α-CoV sequence from a frugivorous *L. angolensis* clustered with CoV sequences mainly obtained from insectivorous Molossidae bats from Cameroon, Tanzania, Southern Africa and the Western Indian ocean.

β-CoVs were observed in 18/274 (6.6%) frugivorous bats and 3/45 (4.4%) insectivorous bats. None of the new viruses was closely related to the human β-CoVs (Figure 2B). Among β-CoVs, our sequences fell into three subgenera, recently named *Sarbecovirus*, *Merbecovirus* and *Nobecovirus* [50]. All the 18 β-CoV sequences detected in fruit bats clustered in the NobeCoV subgenus comprising only CoVs from Pteropodid bats from various places in Asia and Africa (i.e., Kenya, Cameroon, Rwanda, Madagascar, Congo, Tanzania). New β-CoVs strains from *E. helvum* clustered with previously reported CoV strains form this species observed in bats from Cameroon, Kenya and even Saudi Arabia. However, a β-CoV from a *Rousettus* bat from Madagascar fell also in this clade. Similarly, the five new β-CoV strains from *Epomophorus* bats clustered with previously documented strains from this genus from other regions in Africa, but strains from other bat species like *Micropteropus pusillus* or *Rousettus* sp. from central and Eastern Africa were also observed in this clade. Interestingly, the new β-CoV from the *Nanonycteris* bat in Guinea fell in the same cluster. The new strains from *Lissonycteris angolensis* were closely related to a recently reported β-CoV from the same species in Rwanda. Among the new β-CoVs from *Rousettus aegyptiacus,* two strains formed a separate lineage with no close relatives (>90% homology) in Genbank and the four remaining strains clustered with β-CoVs identified in *Rousettus* sp. from Cameroon or Kenya.

The β-CoVs observed in insectivorous bats belonged to two subgenera. The two β-CoV strains from *Hipposideros* bats belonged to the SarbeCoVs subgenus, that also harbor the human SARS-CoV strains as well as other closely related CoVs from insectivorous bats, civets and pangolins. Our sequences were highly diverse and fell in a clade grouping a wide diversity of CoVs, all from *Hipposideros* bats in diverse African countries. Finally, the new β-CoV strain from the *Nycteris* bat, trapped in a cave, belongs to the MerbeCoV genus which includes the CoVs responsible for the human Middle East respiratory syndrome and closely related CoVs detected in camels and Vespertilionidae bats (*Pipistrellus* sp. from China, Uganda and *Neoromica* sp. from South Africa). Our sequence clustered specifically in a distinct clade with other strains from *Nycteris* bats from Mozambique and Ghana that was basal to the MERS-CoV related viruses.

Overall, *Rousettus* and *Hipposideros* bats from our study are infected with at least three different CoVs (one α-CoV and two β-CoVs) and *L. angolensis* bats with α-CoV and β-CoVs.

## 4. Discussion

With the recent emergence of the COVID-19 pandemic caused by SARS-CoV-2 and the previous highly pathogenic SARS-CoV-1 and MERS-CoV outbreaks, it is important to better document the diversity and evolution of coronaviruses, particularly in regions at high-risk for emergence of infectious diseases of zoonotic origin. All CoVs in humans have a zoonotic origin with a direct or indirect link with bat CoVs. Here we documented for the first time the presence and genetic diversity of alpha and beta coronaviruses in bats in Guinea, West Africa, where contacts between humans and bats are frequent. In this pilot study, we analyzed more than 300 bats from at least eight frugivorous and six insectivorous species, collected at 14 different areas in seven prefectures across the country. We detected and characterized coronaviruses in 11% of the bats, at almost all sites and observed a wide diversity of α and β-CoVs with potential new viral lineages. Some strains belonged to the subgenera of MerbeCoV and SarbeCoVs known to harbor highly pathogenic HCoVs like MERS-CoV and SARS-CoV, respectively. We also report for the first time, the presence of a coronavirus in a bat from the genus *Nanonycteris*.

The overall rate of CoV detection (11%) in our study is consistent with the rates found in previous studies in Kenya and Mozambique [40,51], but infection rates in studies in central and southern Africa and the western Indian Ocean were generally lower [15,35,38,39,40,42,52]. Positivity rates of coronavirus detection can vary by species and season, therefore comparison between studies is limited. In Guinea, we also observed differences in positivity rates according to species. Although we tested only a few samples per species, high rates were observed in three insectivorous genera from three families (7/21 *Hipposideros* sp., 1/3 *Nycteris* sp. and 2/5 *Rhinolophus* sp.) and two frugivorous species (4/9 *Eidolon helvum* and 1/1 *Nanonycteris* sp.). However, it cannot be excluded that this could be related to the season when samples were collected, for example several studies have shown temporal variations of CoV infections associated with parturition [37,41,53,54,55]. Longer follow-up should be done on these bat species in Guinea to assess temporal dynamics of CoV infection. Most samples have been obtained in cities or villages close to human habitats, it is thus important to know whether exposure to bat CoVs vary during certain periods of the year. Differences in positivity rates can also be explained by the nature of samples that have been tested, for example in our study we observed higher rates in feces and rectal swabs than in saliva. This was also observed in other reports and confirms that coronaviruses are highly shed in the intestinal tract [56]. Therefore, non-invasive collection of fresh bat feces, under roosting or feeding sites, may be more efficient to study CoVs, especially for species that are difficult to access.

Phylogenetic analyses revealed a wide diversity of α and β-CoVs with potential examples of co-evolution between CoVs and their bat hosts and examples of host diversity within CoV lineages. The genetic diversity observed in our study is most likely associated with the high diversity of bat species tested, which is in accordance with previous findings [40]. It also confirms the observations from the large study on CoVs in more than 12,000 bats across Africa and Asia from Anthony et al. (2017) who demonstrated that increasing sampling efforts leads to increased identification of new lineages. Although we studied only very few samples from *Hipposideros* bats, we did not only find a high infection rate (7/21, 33%), but observed also a high genetic diversity among their CoVs in accordance with most previous studies on *Hipposideros* species in Africa [15,34,35,39,40,41,42]. The human 229E α-CoV falls in a clade of bat CoVs that is widely present in *Hipposideros* bats from Kenya, Zimbabwe, Mozambique, Gabon, Ghana and also in our study in Guinea. Hipposiderids roost in hollow trees, small caves and abandoned buildings, and indirect and direct exposure to infected bats and to the wide diversity of CoVs is thus very likely [57,58]. Moreover, the β-CoVs observed in *Hipposideros* bats belong to the SarbeCoV subgenus where they form a sister-clade to the clade of human and animal CoVs related to SARS-CoV-1 and -2. Within this viral clade, the genetic diversity is also very high, and viruses from this clade are present in *Hipposideros* species across Africa [15,35,39,41,59]. CoVs have also been described in *Hipposideros* bats in China and Thailand, suggesting a potential new clade [55,60,61]. The β-CoV observed in the *Nycteris* bat from Guinea falls with other CoVs from *Nycteris* bats in the MerbeCoV subgenus, forming a sister clade to the MERS-CoV related viruses from humans and animals. This clade is also widespread in *Nycteris* species across Africa [37,40]. All β-CoVs from frugivorous bats fall within the NobeCoV subgenus, in which for certain species potential new viral lineages are seen. However, this could be related to the low number of CoVs available among GenBank references. For α-CoVs we also observed mainly association between CoVs and their bat hosts, except for α-CoV virus identified in the frugivorous *L. angolensis* bat (CCGU550) clustering with α-CoVs from insectivorous bats.

As in other studies, we also showed that the same bat species can be infected with different α and/or β-CoV lineages and species; this was seen in *L. angolensis*, *R. aegyptiacus* and *Hipposideros* sp. bats [15,34,39,51,56,62]. We observed a co-infection in a single *Rousettus aegyptiacus* bat with an alpha and betacoronavirus. Co-infections have been previously reported among insectivorous bats in Asia, but not yet in Africa [61,62,63].

We confirm that different viruses co-circulate in the same species and that certain viral strains may be maintained in more than one host and that cross-species transmission may occur, as previously reported [34,64]. These results are important because viral sharing and cross-species transmission may lead to recombination events and the emergence of novel bat coronaviruses or novel coronaviruses in other mammal species naturally infected with CoVs [64,65]. Recent studies demonstrated that frequent cross-species transmission seem to explain the evolutionary history of CoVs rather than co-divergence [56,66]. Moreover, viruses with a higher taxonomically and ecologically diverse host range seem to be more likely to adapt to humans and spread on a broad geographic scale and thus with a higher pandemic potential [65].

With this study in Guinea, we brought additional knowledge on coronaviruses in bats in Africa and especially in West Africa where only information was available for bats from Ghana and for small numbers of bats and species in Senegal and Nigeria [15,35,37,59]. The fact that we observed a high genetic diversity with potential new viral variants illustrates clearly that knowledge on bat coronaviruses is still incomplete and that more bat CoVs will be discovered as sampling efforts will increase. Anthony et al. (2017) even estimate that more than 3000 bat CoVs circulate in the more than 1400 known bat species [56]. Given the impact of zoonotic transmissions from CoVs to humans, it is important to document the CoVs that circulate in bats, but also in other wildlife species because for certain HCoVs, intermediate hosts were involved. For example, the closest relative to SARS-CoV-2 is the SARSr-Ra-BatCoV-RaTG13 from *Rhinolophus* bats from China, sharing more than 96% identity at the whole-genome level, but the receptor binding domain (RBD) exhibits stronger similarity to CoVs identified in pangolins, suggesting recombination [24,25]. However, it cannot be excluded that a not yet identified CoV in bats or another animal species is at the origin of the current SARS-CoV-2 epidemic. A prerequisite for cross-species transmission is compatibility between virus and human receptors, therefore future studies should not only focus on partial RdRp sequences but should thus also include characterization of the RBD of CoVs. In addition, as reminder, our findings are based on a small fragment of the RdRp gene, which is a highly conserved region. Therefore, complete genome sequencing and analysis of some key genes such as the Spike and ORF8 genes that have been shown to be hypervariable, should allow to assess recombination events [61,64]. This will bring additional data to retrieve phylogenetic and temporal relationships between these bat CoVs and other pathogenic strains and thus give us more insight into their zoonotic potential.

## 5. Conclusions

In this pilot study, we have demonstrated high rates and important genetic diversity of CoVs in frugivorous and insectivorous bats in Guinea. More studies are needed on CoVs in bats and wildlife in Guinea, but also in other geographic areas of Africa. This will certainly lead to the identification of more novel CoV species and maybe also new genera. Although we now have data for several thousands of bats worldwide, this still remains relatively small given the high numbers of bats that constitute colonies and the high number of bat species worldwide [67]. Future studies should focus on areas and species where contacts between humans are frequent or could become frequent due to environmental and climate changes. The high genetic diversity and complex evolutionary history of co-evolution, host switching, and recombination illustrates the complexity to track the animal reservoir of CoVs and to predict the areas at highest risk and species infected with CoVs with higher adaptive probability to infect humans. Efforts must continue not only on bats, but also on other animals to elucidate where CoVs circulate in wildlife and to clarify the role of intermediate species.

## Figures and Tables

**Table 1:** Number of bat samples collected at each study site in the different prefectures in Guinea.

Prefecture	Site	Number
**Conakry**	CN	59
	Subtotal	59
**Gueckedou**	GK	6
	TM	41
	Subtotal	47
**Kankan**	BN	8
	KK	12
	Subtotal	20
**K** **i** **ndia**	KN	13
	MO	54
	SG	35
	Subtotal	102
**Koundara**	GN	35
	SA	15
	YK	6
	Subtotal	56
**Macenta**	MA	9
	Subtotal	9
**Mamou**	KR	15
	OK	11
	Subtotal	26
**Total**		319

Sites are indicated with a two-letter code as in Figure 1. Sites are abbreviated as follows: CN—Conakry; KN—Kindia; MO—Madina Oula; SG—Souguéta; KK—Kankan; BN—Baté-Nafadji; TM—Termessadou-Dibo; GK—Tékoulo; GN—Guingan; SA—Sareboido; YK—Youkounkoun; KR—Konkouré; OK—Ouré-Kaba; MA—Macenta.

**Table 2:** Number and percentage of samples positive for coronavirus RNA in different bat families and species collected in each site.

Family *Species*	Conakry		Kankan		Kindia		Koundara		Gueckedou		Macenta		Mamou			
	CN	BN	KK	KN	MO	SG	YK	SA	GN	GK	TM	MA	OK	KR	Total	% pos
**Insectivorous Bats**																
**Hipposideridae**	0/1	1/4	0/1	6/13	0/1	-	0/1	-	-	-	-	-	-	-	7/21	33.3%
*Hipposideros* sp.	0/1	1/4	0/1	6/13	0/1	-	0/1	-	-	-	-	-	-	-	7/21	33.3%
**Molossidae**	-	-	-	-	0/3	-	-	-	0/10	-	-	-	-	-	0/13	0.0%
*Chaerephon* sp.	-	-	-	-	0/3	-	-	-	-	-	-	-	-	-	0/3	0.0%
*Mops condylurus*	-	-	-	-	-	-	-	-	0/10	-	-	-	-	-	0/10	0.0%
**Nycteridae**	-	1/2	-	-	-	-	-	-	0/1	-	-	-	-	-	1/3	33.3%
*Nycteris* sp.	-	1/2	-	-	-	-	-	-	0/1	-	-	-	-	-	1/3	33.3%
**Rhinolophidae**	-	-	2/5	-	-	-	-	-	-	-	-	-	-	-	2/5	40.0%
*Rhinolophus* sp.	-	-	2/5	-	-	-	-	-	-	-	-	-	-	-	2/5	40.0%
**Vespertilionidae**	-	-	-	-	0/2	-	-	-	0/1	-	-	-	-	-	0/3	0.0%
*Scotophilus leucogaster*	-	-	-	-	0/2	-	-	-	0/1	-	-	-	-	-	0/3	0.0%
**Fruit Bats**																
**Pteropodidae**	6/58	0/2	0/6	-	2/48	2/35	1/5	1/15	3/23	1/6	6/41	2/9	0/11	1/15	25/274	9.1%
*Eidolon helvum*	3/6	-	-	-	-	-	-	0/1	-	-	-	1/2	-	-	4/9	44.4%
*Epomophorus gambianus*	3/48	0/2	0/5	-	0/2	-	1/3	1/14	0/2	-	-	-	0/11	-	5/87	5.7%
*Epomops buettikoferi*	-	-	-	-	-	-	-	-	-	0/3	-	-	-	-	0/3	0.0%
*Hypsignathus monstrosus*	-	-	-	-	-	-	-	-	-	0/1	-	0/5	-	-	0/6	0.0%
*Lissonycteris angolensis*	-	-	-	-	0/8	0/6	-	-	2/13	-	-	-	-	1/15	3/42	7.1%
*Micropteropus pusillus*	0/4	-	0/1	-	-	-	-	-	0/1	-	-	-	-	-	0/6	0.0%
*Nanonycteris veldkampii*	-	-	-	-	-	-	-	-	-	1/1	-	-	-	-	1/1	100%
*Rousettus aegyptiacus*	-	-	-	-	2/38	2/29	0/2	-	1/7	0/1	6/41	1/2	-	-	12/120	10.0%
**Total**	6/59	2/8	2/12	6/13	2/54	2/35	1/6	1/15	3/35	1/6	6/41	2/9	0/11	1/15	35/319	11.0%
% pos per site	10.2%	25.0%	16.7%	46.2%	3.7%	5.7%	16.7%	6.7%	8.6%	16.7%	14.6%	22.2%	0.0%	6.7%		

CN—Conakry; KN—Kindia; MO—Madina Oula; SG—Souguéta; KK—Kankan; BN—Baté-Nafadji; TM—Termessadou-Dibo; GK—Tékoulo; GN—Guingan; SA—Sareboido; YK—Youkounkoun; KR—Konkouré; OK—Ouré-Kaba; MA—Macenta.; ’-‘ —not applicable because this species was not captured at that site.

**Table 3:** Detail on bat samples in which coronavirus sequences have been amplified. Sites are abbreviated as in Table 1.

Sample	Collection Date	Prefecture	Site	Environment	Species	Type of Sample			Accession Number
						Rectal Swab	Feces	Oral Swab	
**CCGU00021**	18 February 2016	Conakry	CN	city garden	*Epomophorus gambianus*	β-CoV	na	–	MT586830
**CCGU00033**	21 February 2016	Conakry	CN	city garden	*Epomophorus gambianus*	β-CoV	na	–	MT586831
**CCGU00035**	21 February 2016	Conakry	CN	city garden	*Epomophorus gambianus*	–	β-CoV	–	MT586832
**CCGU00039**	20 April 2016	Conakry	CN	city garden	*Eidolon helvum*	β-CoV	na	–	MT586833
**CCGU00040**	20 April 2016	Conakry	CN	city garden	*Eidolon helvum*	β-CoV	na	–	MT586834
**CCGU00041**	20 April 2016	Conakry	CN	city garden	*Eidolon helvum*	β-CoV	na	–	MT586835
**CCGU00081**	31 May 2016	Kindia	KN	cave/village	*Hipposideros ruber*	na	α-CoV	–	MT586836
**CCGU00082**	31 May 2016	Kindia	KN	cave/village	*Hipposideros ruber*	α-CoV	na	–	MT586837
**CCGU00085 ^1^**	31 May 2016	Kindia	KN	cave/village	*Hipposideros ruber*	na	α-CoV	α-CoV	MT586838; MT586839
**CCGU00086**	31 May 2016	Kindia	KN	cave/village	*Hipposideros ruber*	na	β-CoV	–	MT586840
**CCGU00087 ^1^**	31 May 2016	Kindia	KN	cave/village	*Hipposideros ruber*	α-CoV	na	α-CoV	MT58684; MT586842
**CCGU00090**	31 May 2016	Kindia	KN	cave/village	*Hipposideros ruber*	α-CoV	na	–	MT586843
**CCGU00102**	21 July 2016	Koundara	GN	cave/village	*Lissonycteris angolensis*	β-CoV	na	–	MT586844
**CCGU00104**	21 July 2016	Koundara	GN	cave/village	*Lissonycteris angolensis*	β-CoV	na	–	MT586845
**CCGU00115**	22 July 2016	Koundara	GN	village	*Rousettus aegyptiacus*	–	na	α-CoV	MT586846
**CCGU00137**	24 July 2016	Koundara	YK	village	*Epomophorus gambianus*	β-CoV	na	–	MT586847
**CCGU00142**	27 July 2016	Koundara	SA	village	*Epomophorus gambianus*	β-CoV	na	–	MT586848
**CCGU00168**	22 September 2016	Kankan	KK	cave/forest	*Rhinolophus darlingi*	na	α-CoV	–	MT586849
**CCGU00200**	25 September 2016	Kankan	KK	cave/forest	*Rhinolophus darlingi*	na	α-CoV	–	MT586850
**CCGU00235**	28 September 2016	Kankan	BN	cave	*Nycteris macrotis*	na	β-CoV	–	MT586851
**CCGU00269**	29 September 2016	Kankan	BN	cave	*Hipposideros ruber*	na	β-CoV	–	MT586852
**CCGU00316**	27 October 2016	Kindia	MO	cave/forest	*Rousettus aegyptiacus*	β-CoV	na	–	MT586853
**CCGU00337**	29 October 2016	Kindia	MO	plantation	*Rousettus aegyptiacus*	β-CoV	na	–	MT586854
**CCGU00399**	2 November 2016	Kindia	SG	cave/forest	*Rousettus aegyptiacus*	β-CoV	na	–	MT586855
**CCGU00424**	3 November 2016	Kindia	SG	forest	*Rousettus aegyptiacus*	β-CoV	na	–	MT586856
**CCGU00445**	5 December 2016	Gueckedou	TM	cave/forest	*Rousettus aegyptiacus*	–	na	α-CoV	MT586857
**CCGU00453**	6 December 2016	Gueckedou	TM	village	*Rousettus aegyptiacus*	β-CoV	na	α-CoV	MT586858-586859
**CCGU00458**	6 December 2016	Gueckedou	TM	village	*Rousettus aegyptiacus*	–	na	α-CoV	MT586860
**CCGU00467**	7 December 2016	Gueckedou	TM	village	*Rousettus aegyptiacus*	–	na	α-CoV	MT586861
**CCGU00477**	7 December 2016	Gueckedou	TM	village	*Rousettus aegyptiacus*	α-CoV	na	–	MT586862
**CCGU00481**	7 December 2016	Gueckedou	TM	village	*Rousettus aegyptiacus*	–	na	α-CoV	MT586863
**CCGU00489**	10 December 2016	Gueckedou	GK	village	*Nanonycteris veldkampii*	β-CoV	na	–	MT586864
**CCGU00499**	12 December 2016	Macenta	MA	city	*Rousettus aegyptiacus*	β-CoV	na	–	MT586865
**CCGU00502**	12 December 2016	Macenta	MA	city	*Eidolon helvum*	β-CoV	na	–	MT586866
**CCGU00550**	9 January 2017	Mamou	KR	cave/forest	*Lissonycteris angolensis*	α-CoV	na	–	MT586867

^1^ identical sequences in both swabs; α-CoV—alphacoronavirus; β-CoV—betacoronavirus; na—no sample; –: PCR-negative sample.

## Supplementary Materials

The following are available online at https://www.mdpi.com/1999-4915/12/8/855/s1, Table S1: Details on coronavirus reference sequences used in phylogenetic tree analysis.
