# Supplementary material for: Wide Diversity of Coronaviruses in Frugivorous and Insectivorous Bat Species: A Pilot Study in Guinea, West Africa
Source: Viruses. 2020 Aug 5;12(8):855. doi: 10.3390/v12080855 (PMC7472279; doi:10.3390/v12080855)
Supplement: Supplementary file 1 [file viruses-12-00855-s001.pdf]

viruses MDPI

Supplementary Materials: Wide Diversity of Coronaviruses in Frugivorous and Insectivorous Bat Species: A Pilot Study in Guinea, West Africa

Table S1. Details on coronavirus reference sequences used in phylogenetic tree analysis.

Strain	Country	Year	Host	Accession	Virus type
**BtKY24**	Kenya	2006	*Eidolon helvum*	HQ728482	Beta-CoV
**CMR900**	Cameroon	2013	*Eidolon helvum*	MG693169	Beta-CoV
**BtKY95**	Kenya	2007	*Eidolon helvum*	GU065439	Beta-CoV
**BtKY92**	Kenya	2007	*Eidolon helvum*	GU065436	Beta-CoV
**18930**	Madagascar	2014	*Rousettus madagascariensis*	MN183192	Beta-CoV
**BtKY89**	Kenya	2007	*Eidolon helvum*	GU065433	Beta-CoV
**130403Eh04 (PREDICT)**	Kenya	2013	*Eidolon helvum*	KX285108	Beta-CoV
**BtKY44**	Kenya	2007	*Eidolon helvum*	GU065388	Beta-CoV
**8466-11**	Madagascar	2011	*Eidolon dupreanum*	KF859764	Beta-CoV
**CMR891**	Cameroon	2013	*Eidolon helvum*	MG693171	Beta-CoV
**CMR704**	Cameroon	2013	*Eidolon helvum*	MG693168	Beta-CoV
**CMR705**	Cameroon	2013	*Eidolon helvum*	MG693172	Beta-CoV
**KSA282**	Saudi Arabia	2012	*Eidolon helvum*	MH396479	Beta-CoV
**ECO70332 (PREDICT)**	Cameroon	2013	*Micropterus pusillus*	KX284986	Beta-CoV
**ECO70527 (PREDICT)**	Cameroon	2013	*Epomophorus gambianus*	KX285008	Beta-CoV
**ECO70509 (PREDICT)**	Cameroon	2013	*Epomophorus gambianus*	KX284993	Beta-CoV
**CD115912 (PREDICT)**	DRC	2014	*Micropterus pusillus*	KX285075	Beta-CoV
**CD116103 (PREDICT)**	DRC	2014	*Epomops franqueti*	KX285102	Beta-CoV
**RC1159 (PREDICT)**	Congo	2014	*Eidolon helvum*	KX285513	Beta-CoV
**BtKY56**	Kenya	2007	*Epomophorus labiatus*	GU065400	Beta-CoV
**AATHA (PREDICT)**	Tanzania	2013	*Epomophorus* sp.	KX285299	Beta-CoV
**140403Bt16 (PREDICT)**	Rwanda	2014	*Epomophorus labiatus*	KX285431	Beta-CoV
**130518Bt35 (PREDICT)**	Rwanda	2013	*Rousettus aegyptiacus*	KX285428	Beta-CoV
**130512Bt05 (PREDICT)**	Rwanda	2013	*Myonycteris angolensis*	KX285426	Beta-CoV
**CMR66**	Cameroon	2013	*Eidolon helvum*	MG693170	Beta-CoV
**BtKY27**	Kenya	2006	*Rousettus aegyptiacus*	HQ728484	Alpha-CoV
**BtKY78**	Kenya	2007	*Rousettus aegyptiacus*	GU065422	Beta-CoV
**HKU9-10-1**	China	2006	*Rousettus* sp.	HM211100	Beta-CoV
**HKU9-3**	China	NA	NA	EF065515	Beta-CoV

**GCCDC1_356**	China	2014	*Rousettus leschenaulti*	NC_030886	Beta-CoV
**ANK011F**	Madagascar	2010	*Pteropus rufus*	KF859760	Beta-CoV
**8563-11**	Madagascar	2011	*Pteropus rufus*	KP696744	Beta-CoV
**BJ232**	China	2014	canine	KX432213	Beta-CoV
**HY24**	China	2017	yak	MH810163	Beta-CoV
**PHE**	China	2008	porcine	KY994645	Beta-CoV
**OC43**	China	2010	human	KP198611	Beta-CoV
**OC43LRTI238**	Mexico	2011	human	KX344031	Beta-CoV
**HKU14-3**	Hong Kong	2007	rabbit	JN874560	Beta-CoV
**HKU1-12**	USA	2010	human	KF686346	Beta-CoV
**HKU1**	Hong Kong	NA	human	AY597011	Beta-CoV
**CCK-NC7**	South Africa	2015	*Neoromicia capensis*	MG817490	Beta-CoV
**CDK-NC1**	South Africa	2015	*Neoromicia capensis*	MG205591	Beta-CoV
**SRP-NC3**	South Africa	2014	*Neoromicia capensis*	MG817484	Beta-CoV
**CGR-NC3**	South Africa	2015	*Neoromicia capensis*	MG817486	Beta-CoV
**HKU697**	Burkina Faso	2015	camel	MG923473	Beta-CoV
**Al-Hasa**	Saudi Arabia	2013	human	KF600644	Beta-CoV
**5038**	South Africa	2015	*Neoromicia capensis*	MF593268	Beta-CoV
**PML-PHE**	South Africa	2011	*Neoromicia capensis*	KC869678	Beta-CoV
**PDF-2180**	Uganda	2013	*Pipistrellus hesperidus*	NC_034440	Beta-CoV
**18974**	Mozambique	2015	*Nycteris thebaica*	MN183195	Beta-CoV
**KW2E-F82**	Ghana	2011	*Nycteris* sp.	JX899382	Beta-CoV
**It2**	Italy	2009	*Rhinolophus ferrumequinum*	KC633200	Beta-CoV
**FR0711-B3**	France	2011	*Rhinolophus ferrumequinum*	KC633207	Beta-CoV
**BB99-04**	Belgium	2009	*Rhinolophus euryale*	KC633202	Beta-CoV
**BtKY72**	Kenya	2007	*Rhinolophus* sp.	KY352407	Beta-CoV
**441**	Rwanda	2008	*Rhinolophus clivosus*	JQ649535	Beta-CoV
**Wuhan-Hu-1**	China	2019	human	NC_045512	Beta-CoV
**761203**	France	2020	human		Beta-CoV
**RaTG13**	China	2013	*Rhinolophus affinis*	MN996532	Beta-CoV
**GX-P4L**	China	2017	pangolin	MT040333	Beta-CoV
**Tor2**	Canada	NA	human	AY274119	Beta-CoV
**SZ3**	Hong Kong	2003	civet	AY304486	Beta-CoV
**RsSHC014**	China	2011	*Rhinolophus sinicus*	KC881005	Beta-CoV
**As6526**	China	2014	*Aselliscus stoliczkanus*	KY417142	Beta-CoV
**Rs4874**	China	2013	*Rhinolophus sinicus*	KY417150	Beta-CoV
**HKU3-1**	China	NA	*Rhinolophus* sp.	DQ022305	Beta-CoV

**Rp-Shaanxi2011**	China	2011	*Rhinolophus pusillus*	JX993987	Beta-CoV
**ECO70036 (PREDICT)**	Cameroon	2013	*Hipposideros caffer*	KX284981	Beta-CoV
**13BG0214**	Gabon	2013	*Hipposideros gigas*	MG963186	Beta-CoV
**13GB215**	Gabon	2013	*Hipposideros gigas*	MG963187	Beta-CoV
**13GB0273**	Gabon	2013	*Hipposideros gigas*	MG963188	Beta-CoV
**292**	Gabon	2009	*Hipposideros caffer*	JX174638	Beta-CoV
**CS105**	Gabon	2009	*Hipposideros ruber*	MG963190	Alpha-CoV
**390**	Gabon	2009	*Hipposideros caffer*	JX174639	Alpha-CoV
**09GB0376**	Gabon	2009	*Hipposideros ruber*	MG963196	Alpha-CoV
**09GB0379**	Gabon	2009	*Hipposideros ruber*	MG963197	Alpha-CoV
**10GB0318**	Gabon	2010	*Hipposideros ruber*	MG963201	Alpha-CoV
**10GB0354**	Gabon	2010	*Hipposideros gigas*	MG963200	Alpha-CoV
**Kwam31**	Ghana	2008	*Hipposideros* sp.	FJ710049	Beta-CoV
**19020**	Mozambique	2015	*Rhinolophus rhodesiae*	MN183146	Alpha-CoV
**19030**	Mozambique	2015	*Rhinolophus rhodesiae*	MN183151	Alpha-CoV
**18991**	Mozambique	2015	*Rhinolophus lobatus*	MN183155	Alpha-CoV
**BtKY83**	Kenya	2007	*Rhinolophus* sp.	GU065427	Alpha-CoV
**19217**	Mozambique	2015	*Mops condylurus*	MN183182	Alpha-CoV
**AATBM (PREDICT)**	Tanzania	2013	*Tadarida* sp.	KX285348	Alpha-CoV
**19207**	Mozambique	2015	*Mops condylurus*	MN183181	Alpha-CoV
**DC84**	South Africa	2016	*Rhinolophus simulator*	MG310234	Alpha-CoV
**AAOSV (PREDICT)**	Tanzania	2012	*Chaerephon pumilus*	KX285262	Alpha-CoV
**ECO05817 (PREDICT)**	Cameroon	2010	*Micropterus pusillus*	KX284952	Alpha-CoV
**ECO70102 (PREDICT)**	Cameroon	2013	*Mops condylurus*	KX284982	Alpha-CoV
**ECO70538 (PREDICT)**	Cameroon	2013	*Mops condylurus*	KX285014	Alpha-CoV
**AATCA (PREDICT)**	Tanzania	2013	*Tadarida* sp.	KX285352	Alpha-CoV
**18238**	Madagascar	2013	*Mormopterus jugularis*	MN183185	Alpha-CoV
**369**	Reunion	2015	*Mormopterus* sp.	MN183188	Alpha-CoV
**004**	Mayotte	2014	*Chaerephon pusillus*	MN183177	Alpha-CoV
**HK140714**	China	2018	*Scotophilus kuhlii*	MN611521	Alpha-CoV
**GX2018A**	China	2017	*Scotophilus kuhlii*	MK211369	Alpha-CoV
**D5.73**	Germany	2007	*Pipistrellus*	EU375869	Alpha-CoV
**HKU6-1**	China	NA	*Myotis* sp.	DQ249224	Alpha-CoV
**SAX2011**	China	2011	*Myotis riketti*	KJ473806	Alpha-CoV
**LUX15**	Luxembourg	2015	*Myotis emarginatus*	KY502383	Alpha-CoV
**Kwam27**	Ghana	2008	*Hipposideros* sp.	FJ710050	Beta-CoV
**Kwam20**	Ghana	2008	*Hipposideros* sp.	FJ710047	Beta-CoV
**Boo348**	Ghana	2008	*Hipposideros* sp.	FJ710043	Beta-CoV
**Kwam19**	Ghana	2008	*Hipposideros* sp.	FJ710046	Alpha-CoV
**Boo344**	Ghana	2008	*Hipposideros* sp.	FJ710044	Alpha-CoV
**KW1C-F161**	Ghana	2010	*Hipposideros ruber*	KT253264	Alpha-CoV

**BtKY229E-8**	Kenya	2010	*Hipposideros vittatus*	KY073748	Alpha-CoV
**229E**	NA	NA	human	AF304460	Alpha-CoV
**18987**	Mozambique	2015	*Hipposideros caffer*	MN183172	Alpha-CoV
**Zaria**	Nigeria	2008	*Hipposideros commersoni*	HQ166910	Beta-CoV
**Zim035Mag**	Zimbabwe	2016	*Hipposideros* sp.	MG000872	Beta-CoV
**Zim019Mab**	Zimbabwe	2016	*Hipposideros* sp.	MG000867	Alpha-CoV
**Zim021Mab**	Zimbabwe	2016	*Hipposideros* sp.	MG000866	Alpha-CoV
**Zim001Mab**	Zimbabwe	2016	*Hipposideros* sp.	MG000865	Alpha-CoV
**Zim037Mab**	Zimbabwe	2016	*Hipposideros* sp.	MG000869	Alpha-CoV
**Zim034Mab**	Zimbabwe	2016	*Hipposideros* sp.	MG000871	Alpha-CoV
**Zim015Mab**	Zimbabwe	2016	*Hipposideros* sp.	MG000866	Alpha-CoV
**Zim040Mab**	Zimbabwe	2016	*Hipposideros* sp.	MG000870	Alpha-CoV
**19174**	Mozambique	2015	*Triaenops afer*	MN183165	Alpha-CoV
**BtKYNL63-15**	Kenya	2008	*Triaenops afer*	KY073746	Alpha-CoV
**GD03**	China	2018	human	MK334044	Alpha-CoV
**NL63**	Kenya	2014	human	KP112154	Alpha-CoV
**BtNv-SC2013**	China	2013	*Nyctalus velutinus*	KJ473809	Alpha-CoV
**GZ151867**	China	2015	*Tylonycteris robustala*	MK720944	Alpha-CoV
**19013**	Mozambique	2015	*Miniopterus mossambicus*	MN183157	Alpha-CoV
**19024**	Mozambique	2015	*Rhinolophus rhodesiae*	MN183159	Alpha-CoV
**BtKY06**	Kenya	2006	*Rousettus aegyptiacus*	HQ728483	Alpha-CoV
**HKU7-1**	China	NA	*Miniopterus magnater*	DQ249226	Alpha-CoV
**CoV100**	Australia	2007	*Rhinolophus megaphyllus*	EU834953	Alpha-CoV
**HKU8-1**	China	NA	*Micropterus pusillus*	DQ249228	Alpha-CoV
**GD2012**	China	2012	*Micropterus fuliginosus*	KJ473797	Alpha-CoV
**19038**	Mozambique	2015	*Rhinolophus rhodesiae*	MN183190	Alpha-CoV
**19029**	Mozambique	2015	*Rhinolophus lobatus*	MN183189	Alpha-CoV
**BCoV-ENT**	US	2001	bovine	AF391541	Beta-CoV
**MHV**	US	2003	*Mus musculus*	FJ647226	Beta-CoV
**PHEV**	Belgium	2005	porcine	DQ011855	Beta-CoV
**PEDV-CV777**	NA	1993	porcine	AF353511	Alpha-CoV
**UU10**	Netherlands	2007	feline	FJ938059	Alpha-CoV
**TGEV-AHHF**	China	2015	porcine	KX499468	Alpha-CoV
**PRCV**	US	2006	porcine	DQ811787	Alpha-CoV
**rodentCoV-JC34**	China	2011	*Apodemus chevrieri*	NC_034972	Alpha-CoV
**FIPV**	US	2011	feline	KC461237	Alpha-CoV
**RatCoV**	China	2013	rodent	KF294380	Alpha-CoV

NA: Non Applicable (missing data).
